# Preliminary Results on the Efficacy of Gel Microencapsulated Acaricides in the Control of Tick Infestations in Dairy Cows and Their Impact on Milk Yield

**DOI:** 10.3390/ani16071075

**Published:** 2026-04-01

**Authors:** Anna K. Kucharska, Stanisław Kościelny, Jerzy Kowal, Stanisław Łapiński, Anna Wyrobisz-Papiewska, Michał Patrzałek, Marcin W. Lis

**Affiliations:** 1 Department of Zoology and Animal Welfare, Faculty of Animal Sciences, University of Agriculture in Krakow, Al. Mickiewicza 24/28, 30-059 Krakow, Polandmarcin.lis@urk.edu.pl (M.W.L.); 2 Department of Research and Development, ICB Pharma Jaworzno, 43-603 Jaworzno, Poland

**Keywords:** acaricide efficacy, α-cypermethrin, cattle ticks, *Ixodes ricinus*, milk yield, permethrin, tick-borne disease prevention

## Abstract

The castor bean tick (*Ixodes ricinus*) is a widespread European ectoparasite and vector of multiple diseases that can impair cattle health and productivity. This study evaluated whether a single application of a gel microencapsulated acaricide (α-cypermethrin and permethrin) reduces *I. ricinus* infestation in dairy cows and affects milk yield. Twenty cows were allocated to treated and control groups; treated animals received one spray at the start of the grazing season, and tick counts were recorded daily for 196 days with monthly milk-yield measurements. Two activity peaks were observed (June and September–October) with reduced abundance in July–August; all control cows were parasitised, with daily counts of 1–18 ticks (median = 2). During the first month after treatment, the acaricide reduced tick numbers by around 80% versus controls (*p* < 0.001); efficacy declined over the ensuing months, and differences were negligible after about five months. No adverse effect on milk yield was detected (*p* = 0.38), and seasonal variation (*p* < 0.0001) and lactation stage (*p* < 0.001) were the primary determinants of production. Primiparous and young cows, as well as cows in mid-to-late lactation, tended to show higher levels of tick infestation. A single application of the gel microencapsulated product provided a significant reduction in tick burden during the first month after treatment in grazing dairy cows, without a negative impact on milk production, supporting its use in endemic areas.

## Simple Summary

Ticks are common parasites that harm grazing cattle in areas with heavy tick infestations by causing restless animals, reduced feed intake, and lower milk production, so practical ways to protect herds are needed. This study tested whether a single spray of a gel containing tiny capsules of two tick-killing chemicals, alpha-cypermethrin and permethrin, could reduce tick numbers on dairy cows and affect milk yield. Twenty cows were split into treated and untreated groups and monitored for 196 days with daily tick counts and monthly milk records. Two seasonal peaks of tick activity were observed (early summer and early autumn), and fewer ticks appeared during the hottest months. In the treated cows, tick numbers fell significantly by about 80 percent in the first month compared with controls, with protection being non-significant after one month and declining gradually to become minimal after five months. The treatment did not reduce milk production. Primiparous cows, young mothers, and cows in mid-to-late lactation were more prone to infestation. A single application of the gel provided a significant, month-long reduction in tick burden and could help farmers protect cattle in areas where ticks are common.

## 1. Introduction

The castor bean tick (*Ixodes ricinus*) is a polyxenous parasite that is non-selective concerning host species, although it demonstrates a preference for homeothermic organisms. All developmental stages may parasitise more than 300 species of birds, reptiles, and mammals, including humans, as well as domestic and farm animals [1,2,3,4]. Numerous studies have focused on the distribution of ticks in forest habitats typical of *Ixodes ricinus*; however, other environments may also harbour abundant tick populations—i.e., grazed pastures that represent contact areas between wildlife, livestock, and humans [5,6,7]. Such pastures play an important role in the epizootiology of tick-borne diseases [8]. Ticks transmit numerous pathogens, including viruses of the genus *Flavivirus*; bacteria such as *Borrelia burgdorferi*, *Anaplasma phagocytophilum*, *Coxiella* spp., and *Rickettsia* spp.; and protozoa of the genera *Babesia* and *Theileria* [9,10,11,12,13,14]. Severe tick infestations in livestock induce restlessness, shorten feeding, and resting times and consequently reduce productivity. They may also lead to anaemia, as a single parasite can ingest approximately 3 cm^3^ of blood during feeding—representing a substantial amount [15,16].

To protect companion animals against ticks, repellents and insecticidal collars are commonly used. In cattle breeding, natural methods are often applied, such as the drainage of wet meadows and pastures and the removal of shrubs [9]. However, chemical methods remain the most common approach, with cattle protected using repellent formulations in the form of baths, sprays, or spot-on applications [17,18]. The method of spraying the vegetation and wild animals that serve as parasite hosts (e.g., deer and rodents) with acaricidal preparations is regarded as the most effective method of tick control [19]. Although α-cypermethrin and permethrin are widely used acaricides, the novelty of the present study lies in the use of a gel microencapsulated formulation, designed to prolong the persistence of active substances on the hair coat and to ensure a gradual release over time. To our knowledge, field data on the long-term field efficacy of microencapsulated acaricides in grazing dairy cattle are still very limited.

The present study was intended to assess the efficacy of a gel microencapsulated acaricide at (1) reducing *Ixodes ricinus* infestations in Polish Red and White dairy cow breeds and (2) to verify whether the application of the preparation affects the milk yield and presence of pyrethroid residues in milk.

## 2. Materials and Methods

### 2.1. Study Area

The study was conducted on a farm located in the village of Czarna (Czarna, Lesser Poland Voivodeship, Poland, 49°31′24″ N, 21°04′57″ E). The farm, established in 1947, covers an area of 20 ha, including 15 ha of meadows and pastures and 5 ha of arable land. The soils are predominantly of medium and low quality (classes IV and V according to the Polish soil classification) [20]. The grasslands are situated in hilly terrain. The farm specialises in the breeding of Polish Red and White cows under a tie-stall/pasture-based management system. The herd, consisting of 20 breeding cows, is included in the conservation program for native breeds, serving as a genetic reserve. During the grazing season, dairy cows, pregnant heifers, and calves remain on pasture 24 h a day. Milking is carried out in the cowshed two times a day-at 05:00 a.m. and 5:00 p.m.

### 2.2. Animals Included in the Study

A total of 20 cows were included in the study (10 control and 10 experimental), and the same animals were monitored repeatedly throughout the grazing season. Cows were randomly allocated to the experimental and control groups based on their ear tag numbers. Each group included older cows (15–20 years of age), middle-aged cows (9–14 years of age), young cows (3–8 years of age) and primiparous individuals as well as dry cows and those in the early- (1–2 months), mid- (3–8 months), late (9–10 months) stages of lactation. The distribution of cows among age categories was as follows: primiparous (*n* = 1), young (3–8 years; *n* = 3), middle-aged (9–14 years; *n* = 3), and old (15–20 years; *n* = 3). Lactation stages were represented by a varied number of cows, depending on the month: dry cows (*n* = 0–5), early lactation (*n* = 0–6), mid-lactation (*n* = 2–8), and late lactation (*n* = 0–3).

Across all monthly observations (*n* = 70), cows were classified as follows: primiparous (*n* = 7), young (*n* = 21), middle-aged (*n* = 21), and old (*n* = 21). Lactation stages across observations were distributed as dry cows (*n* = 17), early lactation (*n* = 15), mid-lactation (*n* = 30), and late lactation (*n* = 8). The numbers reported for age group and lactation stage, therefore, refer to repeated monthly observations of these same cows rather than to independent animals.

Animals from both groups (control and experimental) were always grazed together on all four pastures as part of the standard herd rotation and the farm management routine. No cross-contamination of the preparation was observed during the study. Throughout the entire experimental period, all cows received the same feeding ration. Animals in both groups were comparable in milk yield, age, and stage of lactation. As a consequence, the average daily milk yield during the pasture season in the year preceding the study was 12.1 ± 3.68 kg in the control group and 12.9 ± 3.68 kg in the experimental group, respectively. The selection of individuals was based on the official data from the national system for evaluating dairy cattle performance (Results Report RW-11, by The Polish Federation of Cattle Breeders and Dairy Producers). These data included milk yield (milk quantity, fat, protein, and lactose content, as well as somatic cell count), physiological status, and the course of lactation.

### 2.3. Tick Inventory on Pastures

**Figure A1:**
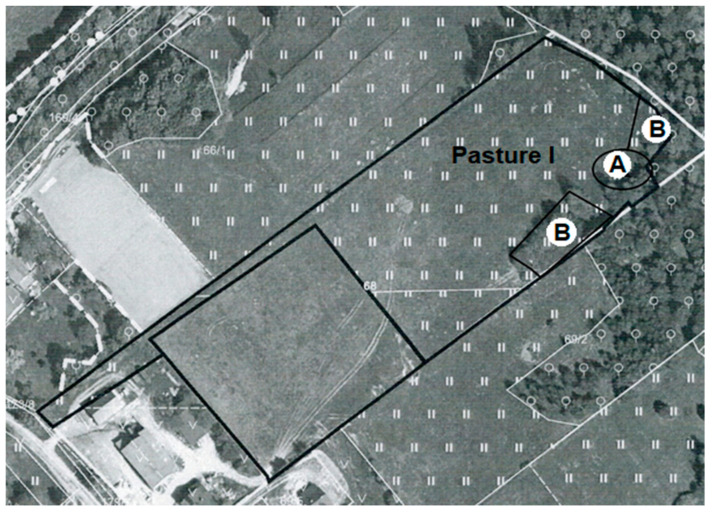
Pasture I is located on a south-facing slope adjacent to a forest. The areas marked “A” and “B” indicate sites where tick habitats were found. The background map shows land cover type. Photo: Pasture map (ARiMR).

**Figure A2:**
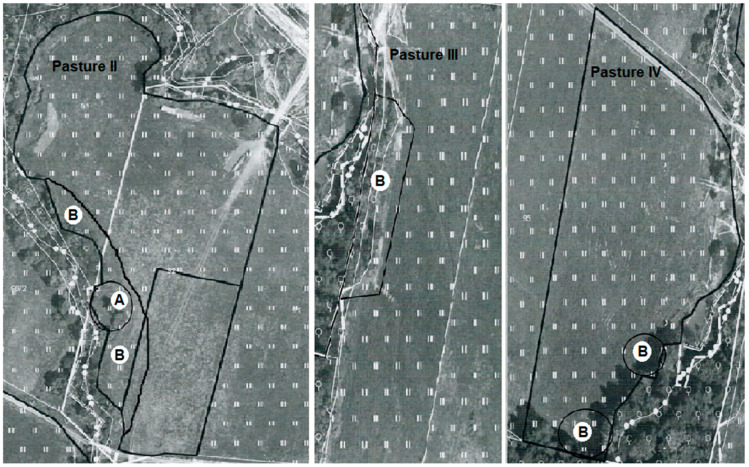
Pastures II, III, and IV are located on a north-facing slope with numerous trees along their edges and adjacent to a natural watercourse. The areas marked “A” and “B” indicate sites where tick habitats were found. A—inspection on 13 April; B—inspection on 15 May. The background map shows land cover types, Photo: Pasture map (ARiMR).

Before testing the acaricidal preparation, an inventory of ticks was conducted to confirm the presence of the castor bean tick (*Ixodes ricinus*). The flagging method was used, with two inspections performed one month apart. The first inspection took place on 13 April—it began at 3:00 p.m. on the southern slope pastures (Figure A1) and ended at 6:00 p.m. on the northern slope pastures (Figure A2). In the afternoon, the temperature on sun-exposed slopes decreased, which favoured tick activity and their movement onto grass blades in search of hosts. Collected arthropods were counted, their numbers and locations were recorded on pasture maps, and the specimens were preserved in ethanol. Pasture surveys were conducted in April and May to characterize the initial tick pressure at the beginning of the grazing season, when cows were first exposed to pasture infestation. Subsequent monitoring focused on tick infestation directly on cows, which reflects the biologically relevant exposure during the entire season.

### 2.4. Inventory of Ticks Parasitising Dairy Cows

The number of ticks on individual cows in the control and experimental groups was monitored daily for the entire grazing period (196 days, from the application of the preparation on 15 May to the cows’ removal from pasture on 26 November). Tick counts were performed in the evening during milking by visual inspection of the entire body surface, with particular attention to preferred attachment sites (inguinal region, udder area, shoulders, neck, and flanks). All inspections were carried out by the same trained observer throughout the study to minimize observer-related variability. Additionally, all ticks present on the animals (unfed and engorged) were collected at weekly intervals. The collected specimens were placed in tubes labelled with the date and the cow’s identification number. Each tick was morphologically identified (all specimens were classified as *Ixodes ricinus*) and subjected to biometric measurements to confirm species identity. Ambient temperature and weather conditions were also recorded daily. The person performing the inspections used protective clothing and gloves to prevent tick transfer to humans.

### 2.5. Evaluation of Milk Performance

Milk yield was assessed at 4-week intervals using the AT-4 method and recorded in RW-11 by authorised staff of the Polish Federation of Cattle Breeders and Dairy Farmers. The data from test milkings were used to compare milk production between the control and experimental groups before and after the preparation’s application.

### 2.6. Tested Preparation

The tested preparation contained the active substances α-cypermethrin and permethrin, encapsulated in gel microcapsules (Table 1). The product was manufactured and supplied by ICB Pharma (Jaworzno, Poland). Immediately before use, a 25% working solution was prepared by diluting the concentrate with water at a ratio of 1:4. The preparation was applied only once, at the beginning of the grazing season (15 May). No re-application was performed during the study to assess the duration of a single treatment. Each cow in the experimental group was sprayed with 50 mL of the prepared solution over the entire hair-covered body surface (avoiding the eyes, nostrils, muzzle area, udder, vulva, and anus to prevent irritation). The preparation was applied to the experimental cows at approximately 9:00 p.m., after evening milking, while the animals were secured in tie stalls. The cows were released to pasture only after the preparation had dried completely, at approximately 07:00 a.m. the following morning. Thus, in accordance with the manufacturer’s recommendations, the animals were turned out to pasture approximately 10 h after treatment. Control group animals were not treated with the preparation. Before application, all cows in both groups were brushed to remove coat dirt and contaminants from the skin surface.

### 2.7. Alpha-Cypermethrin and Permethrin: Regulatory Status and Safety Considerations for Use in Animal and Food-Related Environments

Alpha-cypermethrin and permethrin are synthetic pyrethroid insecticides extensively employed in veterinary and agri-food sectors for the control of ectoparasites and flying insects. They are considered safe and effective for controlled use in animal husbandry and food-related environments when applied in accordance with their regulatory approvals. Both substances are regulated under the European Union Regulation (EU) No 528/2012 concerning the making available on the market and use of biocidal products (BPR) and authorized in accordance with Regulation (EU) 2019/6 on veterinary medicinal products as ingredients of topical formulations (i.e., pour-on, spot-on, and spray applications) for livestock and companion animals.

Alpha-cypermethrin is approved as an active substance for Product Type 18 (PT18; insecticides, acaricides, and products to control other arthropods) pursuant to Commission Implementing Regulation (EU) No 736/2013. Permethrin is approved for Product Types 18 and 19 (PT18–19; insecticides and repellents) under Commission Implementing Regulation (EU) No 703/2013.

### 2.8. Analysis for the Presence of Pyrethroids in Milk Samples

Milk samples were randomly selected from the experimental group two weeks before and two weeks after the preparation was administered. The samples were analysed for the presence of the preparation’s active ingredients (pyrethroids) using gas chromatography with electron-capture detection at the accredited laboratory of the Department of Pharmacology and Toxicology, National Veterinary Institute, Puławy, Poland.

### 2.9. Statistical Analyses

Differences in tick infestation intensity between the control and experimental groups were analysed separately for each month (May–November) to evaluate seasonal changes in treatment efficacy and infestation patterns. Two types of generalized linear mixed models (GLMMs) were fitted: (1) binomial GLMMs for tick presence (0/1), (2) count GLMMs for the number of ticks on infested days (when tick presence was >0).

For the binomial models, tick presence was analysed using a logit link and a binomial error distribution. Fixed effects included treatment (control vs. experimental), lactation stage (dry, early, mid, late), and age category (primiparous, young, middle-aged, and old cows). Cow identity (ID) was included as a random factor to account for repeated daily observations. All biologically plausible two-way interactions were tested and retained only when statistically significant (*p* < 0.05).

For tick counts, GLMMs were fitted using a Poisson distribution with a log link, and when overdispersion was detected, a negative binomial model was used instead. The same fixed and random effects structure as in the binomial models was applied. Count models were fitted only to records with at least one tick found on a cow.

In two months (July and November), all tick-positive observations consisted of only one tick, resulting in no variance in counts. Therefore, the GLMM for tick-count could not be fitted in these months, and only descriptive summaries were reported. A total of 20 cows (10 experimental and 10 control) were observed daily. Across all months, the dataset comprised over 3900 daily records, including 1500+ observations with ticks present (exact numbers varied per month).

A linear mixed-effects model was fitted to analyse the effect of the preparation on milk yield, taking into account treatment group (control vs. experimental), lactation group, age, and month as fixed effects, with cow ID specified as a random effect to account for repeated measurements. Least-squares means for each level of the fixed effects were calculated, and pairwise comparisons between means were evaluated using the Tukey–Kramer adjustment for multiple comparisons. The significance of differences between specific factor groups was verified through Tukey’s test. Calculations were performed using SigmaStat for Windows 3.5 (SyStat Software Inc., San Jose, CA, USA) and SAS Enterprise Guide v7.1 (SAS Institute Inc., Cary, NC, USA). Differences were considered statistically significant at *p* < 0.05.

## 3. Results

With this field survey, we identified the nymphs and imago of only one tick species: the castor bean tick (*Ixodes ricinus*). During the April survey, ticks were identified only in pastures I and II, and no ticks were detected in pastures III and IV. In contrast, in May, ticks were recorded in all pastures, including III and IV (Table 2), but they remained confined to pasture edges—areas that were wooded, shaded, and moist. No ticks were detected in the central, strongly sun-exposed sections of the grasslands

**Figure 1:**
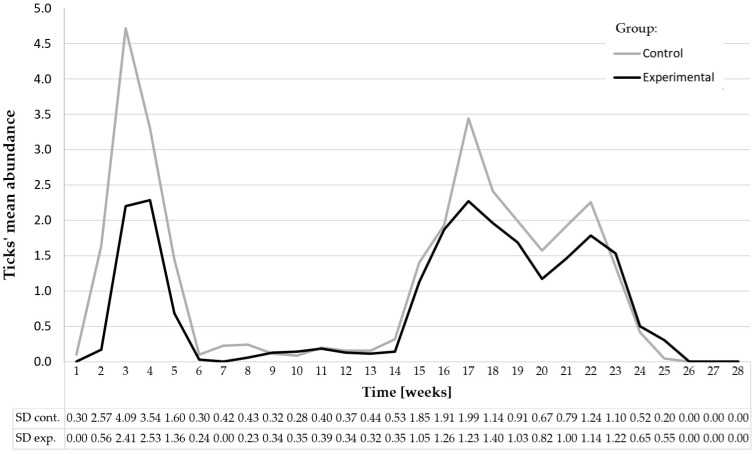
Weekly mean number of ticks (±standard deviations) recorded in the control and experimental groups throughout the entire grazing season (weeks 1–28). Standard deviation values for each week are provided below the graph.

**Figure 2:**
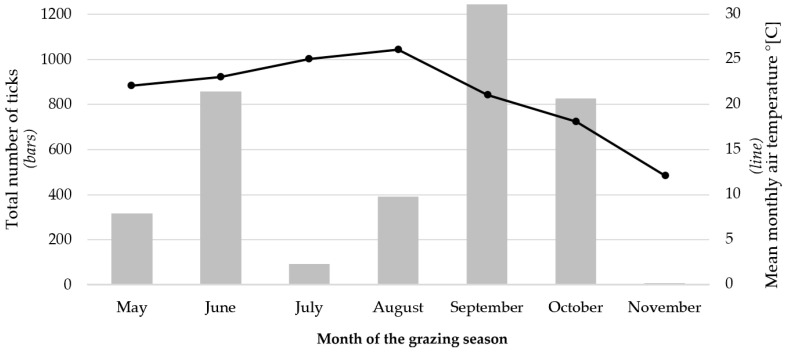
Seasonal variation in infestation of the castor bean tick (*Ixodes ricinus*), and air temperature during the grazing season. Bars represent the total number of ticks recorded during daily inspections of all cows included in the experiment (both groups combined), and the line represents the mean monthly air temperature.

To illustrate the temporal dynamics of tick infestations during the grazing season, the mean number of ticks per cow (±SD) was calculated every week for both groups (Figure 1). This visualisation highlights the seasonal pattern of tick activity and the sustained difference in infestation levels between treated and control cows. During the experiment, two distinct peaks in the abundance of ticks attacking cows were observed: the first was early on in the grazing period (weeks 3–4), and the second was mid-season (approx. weeks 16–23), in September and October, with a clear inter-peak period between July and August (Figure 1, Table 3). In both periods, the control group generally exhibited higher mean tick counts compared with the experimental group. These months were characterised by the highest temperatures (Figure 2), and the precipitation levels were mostly below average for Polish conditions. The mean monthly precipitation during the study period, based on the Meteorological Yearbook published by the Institute of Meteorology and Water Management, was 50.1 mm in May, which increased to 83.2 mm in June and 80.4 mm in July, and then decreased to 50.9 mm in August and 50.2 mm in September. Lower precipitation levels were recorded in October (34.7 mm) and November (14.1 mm). During the remaining weeks, the tick abundance remained low, with minimal differences between groups toward the end of the grazing season (weeks 24–28, Figure 1).

Although seasonal factors strongly influenced tick activity (Figure 2), treatment effects were evaluated via a direct comparison between treated and control cows grazing under identical environmental conditions. In both the control and experimental groups, ticks were found on all examined cows. The daily infestation intensity in the control group ranged from 1 to 18 ticks, whereas in the group protected with the preparation, the number of parasites per day ranged from 1 to 9. Ticks were most frequently located on the skin in the inguinal (flank) and shoulder region.

**Figure 3:**
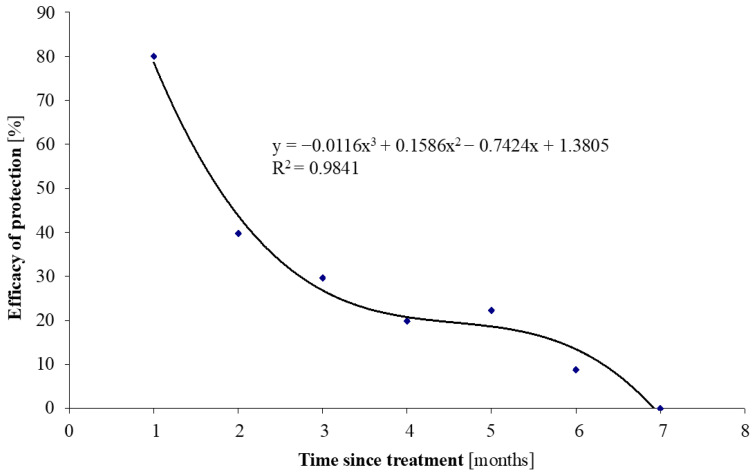
Efficacy of cow protection against tick infestation in subsequent months following the application of the preparation. Blue symbols represent the experimental data points, and the line represents the fitted third-degree polynomial regression model. The decline in the product’s efficacy over subsequent months is well illustrated by the line and third-degree polynomial regression equation, where y = efficacy of the preparation (%), x = month following application.

The application of the preparation resulted in a significant reduction in feeding ticks in May (*p* < 0.009; Table 4). In the following months, the differences between the groups diminished, and from June to October, the infestation intensity did not differ significantly between the control and experimental cows (Table 3). Moreover, the protection efficacy was quantified using a regression equation (Figure 3), which describes the temporal decline in this efficacy over successive months. The efficacy was calculated as E = [(C − T)/C] × 100%, where C is the mean number of ticks in the control group, and T is the mean number in the treated group for a given month.

Moreover, across the study period, the presence and number of ticks on infested days varied substantially by month (Table 4 and Table 5). The effect of the treatment, cow age category, and lactation stage also exhibited clear seasonal patterns.

In May, cows treated with the microencapsulated acaricidal gel had significantly fewer days with ticks compared with control cows (*p* < 0.001; Table 3). Differences between age categories were also noted (*p* = 0.0004): primiparous cows demonstrated the highest likelihood of being infested (mean daily tick presence: 0.40 ± 0.49; Table 4), followed by the old and middle-aged cows, whereas young cows exhibited substantially fewer infestation days (Table 4). The tick infestation intensity varied throughout the lactation period (*p* < 0.001), with dry cows having the lowest probability of carrying ticks, and cows in early or mid-lactation stages exhibiting the highest values. No interaction effects were detected (*p* = 0.29). On days when ticks were present, the number of ticks differed significantly across treatments (*p* = 0.0006), with treated cows having, on average, 80% fewer ticks compared with control animals (5.71 ± 5.85 ticks per cow; Table 5), followed by old and young cows, whereas middle-aged cows exhibited fewer infestations. Differences in the number of ticks have also been identified based on age categories (*p* = 0.02), with primiparous cows showing the highest burdens. The lactation stage (*p* = 0.29) did not significantly influence the tick numbers.

In June, cows from the experimental group had fewer infestation days than controls (*p* = 0.01, Table 4). Age influenced infestation (*p* < 0.05, Table 4), with middle-aged and primiparous cows exhibiting higher infestation rates than young cows. The lactation stage was strongly associated with infestation (*p* < 0.001), and dry cows demonstrated the lowest risk; interaction effects were not significant (*p* = 0.11). The tick numbers on infested days (Table 5) were strongly affected by age (*p* = 0.0065), with primiparous cows exhibiting substantially higher tick burdens (mean number of ticks: 3.67 ± 4.71). Neither the use of the preparation (*p* = 0.38) nor the lactation stage (*p* = 0.78) significantly influenced tick numbers. A significant interaction (*p* = 0.02) indicated that the effect of the treatment on the tick burden differed between age groups.

Infestation levels were low in July. The antiparasitic treatment did not significantly influence the tick occurrence (*p* = 0.09). Age differences were pronounced (*p* < 0.001): primiparous cows exhibited the highest probability of having a tick, whereas old cows had almost no ticks (Table 4). The lactation stage also affected infestation (*p* < 0.001), with dry cows demonstrating the lowest values. Because all cows that carried ticks in July had exactly one tick, tick numbers could not be analysed statistically.

The preparation significantly affected the infestation risk in August (*p* = 0.03), with middle-aged cows having the highest risk of infestation (Table 4). The lactation stage (*p* < 0.001) was statistically significant, with cows in mid-lactation showing the greatest susceptibility. Interaction effects were not significant (*p* = 0.11), and tick numbers differed significantly across lactation stages (*p* = 0.002), with cows in the mid lactation stage carrying more ticks than those in the early lactation stage. The treatment (*p* = 0.10) and age (*p* = 0.39) did not significantly influence tick counts. A significant interaction (*p* = 0.01) indicated that treatment effects varied with age or lactation stage.

In September, infestation levels were the highest across the entire study period (Table 3). Cows treated with the preparation had significantly fewer days with ticks than controls (*p* < 0.001). The infestation probability was dependent on the influence of age (*p* < 0.001, Table 4) and the lactation stage (*p* = 0.0005), although almost all cows were infested on most days regardless of category. Interactions were not significant (*p* = 0.94), and tick numbers varied significantly only with age (*p* = 0.04), with middle-aged cows showing slightly lower counts than the other cows. The treatment (*p* = 0.19), lactation stage (*p* = 0.31), and interactions (*p* = 0.23) were not significant predictors of tick numbers.

Treatment did not significantly influence the tick infestation in October (*p* = 0.59); however, age remained a strong predictor (*p* < 0.001), with young cows exhibiting the highest infestation levels and old cows the lowest. The lactation stage also remained significant (*p* < 0.001)—dry cows carried ticks less frequently than cows in early, mid, or late lactation. Interaction effects were significant (*p* < 0.001), and the number of ticks did not differ significantly by treatment (*p* = 0.67), age (*p* = 0.27), or lactation stage (*p* = 0.39), although interactions were significant (*p* = 0.02).

The tick activity was minimal in November, and almost all cows in both groups had no ticks. A tick number analysis was not possible because all positive observations consisted of only a single tick.

**Figure 4:**
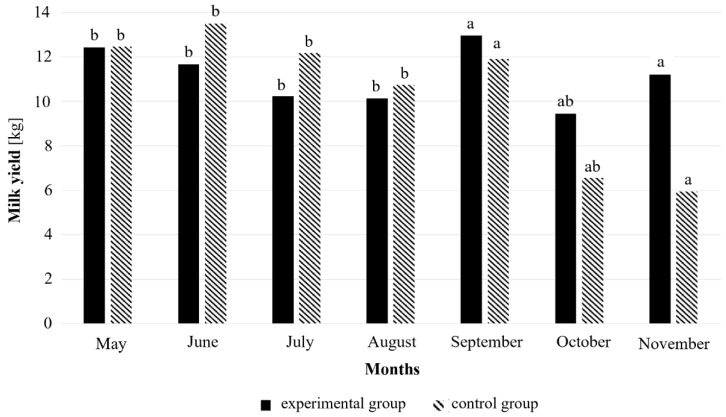
Average milk yield in the experimental and control groups across months of the grazing season. Values represent least-squares means obtained from a linear mixed-effects model with treatment, lactation group, age category, and month included as fixed effects and cow ID as a random effect to account for repeated measurements; ^ab^—different superscript letters indicate significant differences between means according to Tukey–Kramer pairwise comparisons (*p* < 0.05).

During the experiment, the milk yield was significantly affected by the lactation stage (*p* < 0.0001) and month (*p* < 0.0001; Figure 4), whereas no effect was observed for the tick control preparation (*p* = 0.38; Figure 4). The average daily milk yield during the trial milking was 10.2 ± 4.49 kg in the control group and 10.3 ± 4.49 kg in the experimental group (*p* = 0.88). In both groups, the highest milk production was recorded from May to September (10.1–13.5 kg) and decreased in October and November to 5.5–7.0 kg (*p* < 0.05 for seasonal decline). Although the overall effect of the treatment on the milk yield was not statistically significant, from September to November, the experimental group showed numerically higher milk production than controls (Figure 4), indicating a possible short-term beneficial trend. Cows in their early lactation stage produced the most milk (12.82 ± 0.72 kg), followed by those in their mid-stage (7.48 ± 0.61 kg), late stage (4.02 ± 0.89 kg), and dry cows (0.21 ± 0.78 kg). All pairwise comparisons between lactation groups were statistically significant (all *p* < 0.001), and the cows’ age did not significantly affect the milk yield (*p* = 0.45).

No α-cypermethrin or permethrin residues were detected in any of the analysed milk samples either before or two weeks after treatment (limit of detection: 5 µg/kg for both compounds). These results confirm that the applied formulation does not lead to a measurable transfer of pyrethroids into milk.

## 4. Discussion

Among the ectoparasites of ruminants, ticks are of the greatest importance across Europe. In Poland, two species are commonly found—the castor bean tick (*Ixodes ricinus*) and the ornate cow tick (*Dermacentor reticulatus*), which are mainly restricted to the eastern regions of the country [21,22,23]. *I. ricinus* has a wide geographical range covering almost all of Europe, except for the north-eastern areas at the edge of the continent that lack tree cover [24].

In the present study, all specimens collected during the pasture inventories were identified as *I. ricinus*. Tick infestation foci on the investigated pastures were identified based on field surveys conducted by the authors.

These foci were located in areas with tree cover, often near watercourses, whereas no ticks were found in central, strongly sunlit zones. This observation supports the widely accepted view that ticks occur mainly in deciduous and mixed forests, as well as on moist meadows and pasture margins [9].

The abundance of *I. ricinus* in a given habitat is closely related to microclimatic conditions, such as high relative humidity (preferably 80–100%), weak wind, small diurnal temperature fluctuations, and the availability of hosts [25,26,27]. The results of the present study partly confirm these relationships. During periods characterised by a higher relative humidity and weak winds, increased tick activity was recorded, whereas dry weather and stronger winds coincided with reduced infestation levels (field observations; no instrumental wind measurements were available). During the first inspection, conducted in mid-April, a relatively low number of ticks were found, and they were present mainly on the south-facing pasture. This may have been related to the still low night-time temperatures and the limited regrowth of the sward at that time. In contrast, the survey carried out one month later (in May) revealed tick foci on all pastures examined, undoubtedly associated with rising air and soil temperatures as well as the high humidity caused by frequent May thunderstorms. Seasonal variation clearly shaped the overall infestation dynamics; however, the comparison between treated and untreated cows grazing simultaneously under the same conditions indicates that the early reduction observed in May was attributable primarily to the acaricide application rather than to the season alone.

During this study, two tick infestation peaks were observed: the first was in June, and the second was in September/October. This pattern is consistent with the ecology of ticks [28] and can be explained by the mass appearance of these arachnids in periods of high humidity and moderate temperatures. During seasonal tick invasion peaks, preventive actions should be employed to protect cattle and mitigate the negative effects of parasitism. The use of pyrethroid-based preparations usually provides satisfactory results [29], and their application does not pose a risk to milk quality [30].

The application of acaricides appears to be the most effective method of tick control [29,31,32,33]. In contrast to conventional sprays and pour-on formulations, which provide only short-lived killing and repellent activity, the microencapsulated gel used in the present work represents a technological advancement that enables slow release and prolonged protection. This formulation demonstrated high efficacy, achieving an approximately 80% tick reduction during the first month after application, followed by a gradual decline in protective effects over subsequent months.

With treated cows, we observed that ticks were mainly located on skin areas exposed to mechanical abrasion during resting, rubbing against objects, or contact with other animals. Interestingly, the highest numbers of ticks, regardless of group, were recorded in primiparous cows. This likely results from their lower social rank within the herd; younger cows are the last to access the best grazing areas [34] and are thus forced to feed on the pasture periphery, where the tick pressure is greater. This finding indicates the need for special protection for first-calving cows. Compared to controls, treated cows had about 25% fewer ticks in the first lactation stage group and about 35–40% fewer ticks in the young (3–8-year-old) group. The reduction in young cows was statistically significant (*p* < 0.05), whereas the reduction in first lactation cows did not reach significance, though this suggests a biologically relevant trend.

The results indicate that the intensity of the tick infestation was significantly affected by the lactation stage. The lowest number of parasites in both groups was identified in early lactation stage cows and dry cows, whereas cows in the late lactation stage exhibited the highest infestation levels. This pattern may be associated with differences in behaviour and the time spent on grazing. Cows with lower milk yields or dry cows typically spend more time resting and less time on pasture, reducing their exposure to ticks. In contrast, cows during the mid- and late stages of lactation have very high energy demands [35]. Consequently, they exploit the available grazing area more intensively (consuming more forage) and, therefore, frequently enter zones where ticks are most abundant.

Tick infestations negatively affect dairy cattle welfare through irritation, blood loss, and increased physiological stress, which may indirectly reduce milk yields [36,37]. Previous studies have demonstrated that moderate to heavy tick burdens are associated with decreased milk production, primarily due to reduced feed intake and the diversion of energy away from lactation [36,38]. Therefore, the milk yield was included as a parameter, although seasonal factors and the lactation stage remain the primary determinants of production performance [37].

Although the preparation effectively reduced the number of ticks on cows, no effect on the test-day milk yield was observed during the study period. Milk production depended primarily on the lactation stage, season, and weather conditions [39,40,41]. Therefore, the acaricide did not negatively influence the milk yield, and even considerable tick infestations at the observed level did not significantly reduce milk production, likely due to the moderate intensity of the infestation and the short feeding duration of ticks on treated animals. Several limitations of the present study should be acknowledged. This experiment was conducted on a single farm and involved a relatively small number of animals; however, the long observation period and intensive repeated measurements allowed for robust mixed-model analyses. The primary aim was to evaluate field efficacy and safety under practical farming conditions rather than to compare different acaricides or formulations. Due to the relatively small number of animals in the experimental groups, the results should be interpreted with caution and considered as indicative rather than definitive.

## 5. Conclusions

The cows’ age was one of the most influential factors throughout this study: primiparous and young cows exhibited both higher infestation probabilities and higher tick burdens. The lactation stage primarily influenced the likelihood of finding ticks, with dry cows demonstrating the lowest risk across all months. Infestation levels exhibited less consistent associations with treatment or lactation and were difficult to interpret in months with very low counts (July and November). Overall, the results demonstrate that the treatment efficacy, cow age, and lactation stage jointly shape seasonal tick infestation patterns.

In conclusion, across the entire grazing season, the application of the gel microencapsulated acaricide displayed the strongest effect during spring and midsummer (May–August), consistently reducing the number of days on which ticks were found. The formulation significantly reduced the infestation intensity of *Ixodes ricinus* in grazing dairy cows by ~80% in May. Its protective effects were observed for up to five months, although statistical significance was limited to the first month after application, reducing tick numbers by ~40% in June, ~30% in July, ~20% in August, ~22% in September, and ~9% in October. Although numerical reductions were observed in subsequent months, these differences were not statistically significant.

The preparation did not exert any significant effect on milk yield, and no differences in production were observed between treated and untreated cows, suggesting that moderate tick infestations did not markedly impair milk performance.

The results indicate that spraying cattle with the gel microcapsule-based acaricide can be an effective method of protecting cows against ticks during the grazing season, without adversely affecting animal health or milk production. Particular attention should be given to primiparous cows, as they appear to be more susceptible to tick attacks and therefore require targeted preventive measures.

## Figures and Tables

**Table 1:** Composition (mass percentage) of the anti-tick preparation, containing active substances enclosed in gel microcapsules (ICB Pharma, Jaworzno, Poland).

No.	Component	% *w*/*w*
1	α-cypermethrin	5.00
2	permethrin	5.00
3	UV protector	2.50
4	hair-binding system	2.50
5	demineralised water	85.00

**Table 2:** An abundance of different developmental stages of the castor bean tick (*Ixodes ricinus*) was observed on the surveyed pastures in April and May.

			Number of Ticks			
Date (Day/Month)	Time	Pasture No.	Females	Males	Nymphs	Total
13 April	3:00 p.m.	I	5	6	7	18
	4:00 p.m.	II	4	7	2	13
	5:00 p.m.	III	0	0	0	0
	6:00 p.m.	IV	0	0	0	0
14 May	3:00 p.m.	I	15	13	14	42
	4:00 p.m.	II	20	9	10	39
	5:00 p.m.	III	17	14	5	36
	6:00 p.m.	IV	18	23	11	52

**Table 3:** Scale of cow infestation by the castor bean tick (*Ixodes ricinus*) after application of the gel microencapsulated pyrethroid preparation during subsequent months of the grazing period. Values represent the sum of ticks recorded during daily visual inspections of all cows within each group during a given month.

Month	Control Group (Untreated Cows)	Experimental Group (Cows Treated with a Microencapsulated Acaricidal Gel)	Reduction vs. Control [%]
	Infestation Intensity [No. of Ticks]		
May	265 ^b^	53 ^a^	80.0
June	536	323	39.7
July	54	38	29.6
August	217	174	19.8
September	700	544	22.3
October	433	395	8.8
November	1	8	-
Total	2206 ^b^	1535 ^a^	30.4

^ab^—values marked with different superscript letters in the rows differ significantly (independent samples *t*-test, two-tailed: *p* < 0.05). Percentage reduction was calculated as the difference between the number of ticks in the control and experimental groups, divided by the number of ticks in the control group, and expressed as a percentage. No reduction value was reported for November due to the very low number of observations, which does not allow for a biologically meaningful interpretation of this result.

**Table 4:** Daily presence of the castor bean tick (*Ixodes ricinus*), expressed as the proportion of observation days on which at least one tick was detected (mean ± SD).

Mean Daily Tick Presence ± SD						
Month	Group	All Cows		Age Group		
			Primiparous	Young	Middle-Aged	Old
			(2 yo)	(3–8 yo)	(9–14 yo)	(15–20 yo)
May	con.	0.82 ± 0.39	0.40 ± 0.49	0.06 ± 0.24	0.35 ± 0.49	0.37 ± 0.48
	exp.	0.29 ± 0.47	0.20 ± 0.40	0.08 ± 0.27	0.06 ± 0.24	0.13 ± 0.34
June	con.	0.53 ± 0.51	0.48 ± 0.50	0.33 ± 0.48	0.57 ± 0.50	0.47 ± 0.50
	exp.	0.53 ± 0.51	0.41 ± 0.49	0.32 ± 0.47	0.22 ± 0.42	0.34 ± 0.47
July	con.	0.16 ± 0.37	0.20 ± 0.40	0.19 ± 0.40	0.00 ± 0.00	0.17 ± 0.38
	exp.	0.28 ± 0.45	0.12 ± 0.32	0.11 ± 0.31	0.00 ± 0.00	0.12 ± 0.33
August	con.	0.39 ± 0.50	0.30 ± 0.46	0.24 ± 0.43	0.65 ± 0.49	0.33 ± 0.47
	exp.	0.35 ± 0.49	0.26 ± 0.44	0.19 ± 0.40	0.54 ± 0.50	0.33 ± 0.47
September	con.	1.00 ± 0.00	0.91 ± 0.29	0.85 ± 0.36	1.00 ± 0.00	0.92 ± 0.28
	exp.	1.00 ± 0.00	0.57 ± 0.50	0.96 ± 0.21	0.89 ± 0.32	0.82 ± 0.38
October	con.	0.84 ± 0.37	0.72 ± 0.45	0.87 ± 0.34	0.61 ± 0.50	0.75 ± 0.43
	exp.	1.00 ± 0.00	0.55 ± 0.5	0.78 ± 0.41	0.56 ± 0.50	0.67 ± 0.47
November	con.	0.00 ± 0.00	0.00 ± 0.00	0.02 ± 0.14	0.00 ± 0.00	0.00 ± 0.06
	exp.	0.08 ± 0.27	0.03 ± 0.16	0.05 ± 0.22	0.00 ± 0.00	0.03 ± 0.17

Values are shown for cows treated with an acaricide gel microcapsule formulation containing pyrethroids (exp.) and untreated cows (con.), separated by age group. “All cows” represents the overall mean for each treatment group within a given month. The values represent raw proportions. Statistical differences between groups were tested using a binomial generalized linear mixed model (GLMM) with treatment, lactation stage, and age category as fixed effects and cow ID as a random effect.

**Table 5:** Number of *Ixodes ricinus* ticks recorded on infested days (mean ± SD) in cows treated with an acaricide gel microcapsule formulation containing pyrethroids (exp.) and in untreated cows (con.), by age group. Only records with at least one tick detected on a cow were included.

Number of Ticks on Infested Days (Mean ± SD)						
Month	Group	All Cows	Age Group			
			Primiparous	Young	Middle-Aged	Old
			(2 yo)	(3–8 yo)	(9–14 yo)	(15–20 yo)
May	con.	1.56 ± 3.02	5.71 ± 5.85	1.35 ± 2.15	0.06 ± 0.24	1.65 ± 3.14
	exp.	0.38 ± 1.17	1.41 ± 2.58	0.25 ± 0.56	0.22 ± 0.83	0.26 ± 0.73
June	con.	1.79 ± 2.84	3.67 ± 4.71	1.92 ± 2.78	0.6 ± 1.11	1.47 ± 2.01
	exp.	1.28 ± 2.10	2.43 ± 3.16	1.19 ± 1.80	1.14 ± 2.16	0.93 ± 1.32
July	con.	0.17 ± 0.38	0.16 ± 0.37	0.20 ± 0.40	0.19 ± 0.40	-
	exp.	0.14 ± 0.34	0.28 ± 0.45	0.12 ± 0.32	0.11 ± 0.31	-
August	con.	0.70 ± 1.39	1.16 ± 1.75	0.58 ± 1.41	0.63 ± 1.27	1.10 ± 0.91
	exp.	0.61 ± 0.95	0.42 ± 0.62	0.53 ± 1.01	0.41 ± 0.94	1.06 ± 0.90
September	con.	2.33 ± 1.49	2.7 ± 1.73	2.48 ± 1.5	1.77 ± 1.38	2.2 ± 1.06
	exp.	1.84 ± 1.22	2.93 ± 1.17	1.23 ± 1.34	2.01 ± 0.89	1.91 ± 1.05
October	con.	1.40 ± 1.18	1.03 ± 0.66	1.48 ± 1.33	1.47 ± 0.95	1.13 ± 0.96
	exp.	1.32 ± 1.11	1.71 ± 0.64	0.92 ± 0.98	1.62 ± 1.16	1.27 ± 1.19
November	con.	-	-	-	-	-
	exp.	-	-	-	-	-

“All cows” represents the overall mean for each treatment group within a given month. The values represent raw means. Statistical differences between groups were tested using a generalized linear mixed model (GLMM) for count data with treatment, lactation stage, and age category as fixed effects and cow ID as a random effect. In July and November, all tick-positive observations consisted of a single tick, resulting in no variance in counts.

## Data Availability

The raw data supporting the conclusions of this article will be made available by the authors on request.

## Appendix A

The maps presented below illustrate the pasture areas where the experimental cows grazed during the study period and where the castor bean tick (*Ixodes ricinus*) surveys were conducted.
